# Editorial: Preventive potential of antioxidants in age-related diseases

**DOI:** 10.3389/fphar.2024.1515004

**Published:** 2024-11-08

**Authors:** Bee Ling Tan, Nattaya Konsue, Lunawati L. Bennett, Ayman El-Meghawry El-Kenawy

**Affiliations:** ^1^ Department of Healthcare Professional, Faculty of Health and Life Sciences, Management and Science University, Shah Alam, Malaysia; ^2^ School of Agro-industry, Mae Fah Luang University, Chiang Rai, Thailand; ^3^ College of Pharmacy, Union University, Jackson, Tennessee, United States; ^4^ Department of Pathology, College of Medicine, Taif University, Al-Hawiyya, Saudi Arabia

**Keywords:** aging, ascorbic acid, flavonoid, oxidative stress, polyphenol, vitamin E, cancer, inflammation

Life expectancy rose from 66.8 years in 2000 to 73.4 years in 2019 (World Health Organization, 2022), reflecting human development, but also introducing new challenges. Aging increases sensitivity to internal and external stimuli, leading to biological and cognitive decline, including psychological impairment, cognitive deterioration, and physical frailty. Oxidative stress, an imbalance between antioxidants and pro-oxidants, is a key contributor to cellular damage and the development of age-related diseases such as osteoporosis, cancer, neurodegenerative disorders, arthritis, diabetes, and cardiovascular disease (Tan et al., 2018). These diseases place a heavy psychological burden on individuals, families, and society, fueling the search for therapeutic agents to prevent premature aging, extends healthspan, and slow degenerative diseases.

Emerging research suggests that curcumin can reduce oxidative stress and enhance immune function (Tan and Norhaizan, 2019). Oxidative damage is closely linked to inherited or acquired defects in enzymes regulating redox signaling pathways. As research progresses, the role of antioxidants holds great promise in the treatment and potential alleviation to combat age-related diseases may become increasingly important. Studies have shown that oxidative stress and obesity-related non-communicable diseases (NCDs) can be alleviated by antioxidant-rich nutrients (Tan and Norhaizan, 2021). Bioactive compounds help protect against oxidative stress and inflammation. This has heightened interest in the antioxidant potential of natural products for preventing age-related diseases.

In the Research Topic “*Preventive potential of antioxidants in age-related diseases*”, 6 articles were published, mainly focusing on plant extract and bioactive metabolites to protect the pathogenesis of age-related diseases. These articles were carefully reviewed and accepted for publication and briefly described as follows.

Lim et al. investigated the effects of the methanol extract of *Ligusticum chuanxiong* Hort. on cognitive deficits induced by bilateral common carotid artery stenosis (BCAS) in mice. Chuanxiong Rhizoma has been considered as ‘*qi* medicine in the blood’ in traditional Asian medicine due to its claimed effects on activating blood circulation. The study demonstrated that Chuanxiong Rhizoma methanol extract can ameliorate cognitive impairments associated with vascular dementia by reducing damage to white matter and modulating the activation of astrocytes and microglia. The research highlighted the potential of Chuanxiong Rhizoma methanol extract as a therapeutic agent for treating ischemic cerebrovascular diseases, particularly in the context of increasing dementia prevalence due to aging populations. The findings suggest that Chuanxiong Rhizoma methanol extract may regulate various genes involved in neurogenesis and inflammatory responses, contributing to its neuroprotective effects. The study suggests that Chuanxiong Rhizoma is effective for the treatment of ischemic cardiovascular disease, including vascular dementia.

Ginseng is a traditional Asian medicine, with anti-aging properties due to its unique bioactive compounds including active peptides, polysaccharides, and saponins. The mechanisms by which dietary intake of ginseng prevents brain damage associated with natural aging remain unknown. Lin et al. used metabolomics, microbiota sequencing, and fecal microbiota transplantation to explore the effectiveness of ginseng in the alleviation of brain aging in mice models. They highlighted ginseng may protect the brain by enriching beneficial bacteria including *Bacteroides*, *Dubosiella*, *Enterobacter*, and *Lachnospiraceae*, and metabolites of S-adenosylmethionine, β-carotene, and ursolic acid by modulating microbiota-gut-brain axis. The study suggests that ginseng could potentially alleviate intestinal aging by enhancing beneficial metabolites and bacteria while decreasing cognitive decline linked to aging via circulation of the beneficial elements in the blood.

Xu et al. reported the potential of dihydromyricetin, a natural flavonoid, to ameliorate bone loss in ovariectomized mice via gut-bone axis. In this study, they investigated the mechanisms underlying the amelioration of bone loss in ovariectomized mice treated with various doses of dihydromyricetin. They discovered that dihydromyricetin may affect osteoporosis in ovariectomized mice by improving bone mineral density and decreasing colonic damage and inflammation levels. Dihydromyricetin may alter gut microbiota composition and increase the production of intestinal short-chain fatty acids, namely, propionate and acetate. They conclude that dihydromyricetin may exert anti-osteoporotic properties via modulation of gut-bone axis signaling.

Huan Shao Dan is a classical traditional chinese medicine prescription that possesses anti-aging effects, mitigates depressive symptoms, and has antioxidant properties. Huan Shao Dan is a polyherbal preparation with *Panax ginseng* C. A. Mey., *Cuscuta australis* R.Br., *Angelica sinensis* (Oliv.) Diels, *Dioscorea opposita* Thunb., *Platycladus orientalis* (L.) Franco, *Plantago asiatica* L., *Psoralea corylifolia* L., *Phellodendron chinense* Schneid., *Cistanche deserticola* Y.C.Ma, *Rehmannia glutinosa* Libosch., *Achyranthes bidentata* Bl., *Polygonum multiflorum* Thunb., and *Schisandra chinensis* (Turcz.) Baill (Wu, 1995). Li et al. used a comprehensive identification approach based on a deep learning model and UPLC-Q Exactive-Orbitrap HRMS to identify the chemical profiles of Huan Shao Dan and explore the potential anti-aging metabolites in Huan Shao Dan. The findings revealed that they are 366 metabolites in Huan Shao Dan, in which 135 metabolites were absorbed into plasma. Several potential anti-aging metabolites were identified in this study including jionoside B1, pseudoginsenoside F11, and ginsenoside Rg1, Rg2, and Rc, suggesting that deep learning analysis may provide valuable biological insights by identifying potential anti-aging metabolites of botanical drugs.

Osteoporosis is the most common form of systemic bone disease, marked by a reduction in bone mass and deterioration of the microstructure of bone tissue, which results in heightened bone fragility. Deng et al. summarized the recent studies of quercetin on the anti-osteoporotic effects in their review article. The study showed that quercetin could improve osteoporosis by decreasing osteoclast activity and differentiation and increasing osteoblast differentiation and activity through modulation of several pathways, for instance, ERK/JNK, OPG/RANKL/RANK, Wnt/β-catenin, and others. The findings discussed in this review suggest that quercetin represents a promising therapeutic and preventive strategy for osteoporosis.

In this Research Topic, 1 opinion paper was published. Matías-Pérez et al. discussed the relationship between dietary antioxidants and age-related macular degeneration. Age-related macular degeneration is a devastating eye disease, characterized by the gradual accumulation of protein and lipid deposits in the retina, resulting in atrophy of the retinal pigment epithelium (RPE), and in certain circumstances, leading to the formation of neovascular membranes that can cause loss of vision and hemorrhages. They reported that the risk of developing age-related macular degeneration can be reduced by consuming a healthy and balanced diet rich in antioxidants, such as brightly colored fruits and vegetables. Furthermore, the intake of processed foods rich in saturated fats and simple sugars should be decreased.

In summary, this Research Topic highlights the key interest as well as the potential of antioxidants in age-related diseases. This Research Topic comprises a diverse research scope that includes identification of chemical profiles, *in vivo* studies, possible mechanisms of action using molecular and network analysis, gut microbiome and metabolomics approach, opinion and review of published antioxidants in age-related macular degeneration and osteoporosis, showed the promising preventive potential of antioxidants in age-related diseases. Several areas remain unaddressed, including detailed research on the specific molecular mechanisms and pathways of plant metabolites in long-term clinical trials and safety profiles. Collectively, this Research Topic highlights the recent advances in antioxidants and potential anti-aging metabolites in age-related diseases including vascular dementia and osteoporosis, and emphasizes the importance of further elucidation to effectively translate the findings into clinical applications.
